# Machine learning-based somatic variant calling in cell-free DNA of metastatic breast cancer patients using large NGS panels

**DOI:** 10.1038/s41598-023-37409-1

**Published:** 2023-06-27

**Authors:** Elisabeth M. Jongbloed, Maurice P. H. M. Jansen, Vanja de Weerd, Jean A. Helmijr, Corine M. Beaufort, Marcel J. T. Reinders, Ronald van Marion, Wilfred F. J. van IJcken, Gabe S. Sonke, Inge R. Konings, Agnes Jager, John W. M. Martens, Saskia M. Wilting, Stavros Makrodimitris

**Affiliations:** 1 Department of Medical Oncology, Erasmus MC Cancer Institute, Erasmus University Medical Center, Rotterdam, The Netherlands; 2 Delft Bioinformatics Lab, Delft University of Technology, Delft, The Netherlands; 3 Department of Pathology, Erasmus MC Cancer Institute, Rotterdam, The Netherlands; 4 Erasmus Center for Biomics, Erasmus University Medical Center Rotterdam, Rotterdam, The Netherlands; 5 Department of Medical Oncology, Netherlands Cancer Institute, Amsterdam, The Netherlands; 6 Department of Medical Oncology, Amsterdam UMC, location Vrije Universiteit Amsterdam, Amsterdam, The Netherlands

**Keywords:** Breast cancer, Next-generation sequencing

## Abstract

Next generation sequencing of cell-free DNA (cfDNA) is a promising method for treatment monitoring and therapy selection in metastatic breast cancer (MBC). However, distinguishing tumor-specific variants from sequencing artefacts and germline variation with low false discovery rate is challenging when using large targeted sequencing panels covering many tumor suppressor genes. To address this, we built a machine learning model to remove false positive variant calls and augmented it with additional filters to ensure selection of tumor-derived variants. We used cfDNA of 70 MBC patients profiled with both the small targeted Oncomine breast panel (Thermofisher) and the much larger Qiaseq Human Breast Cancer Panel (Qiagen). The model was trained on the panels’ common regions using Oncomine hotspot mutations as ground truth. Applied to Qiaseq data, it achieved 35% sensitivity and 36% precision, outperforming basic filtering. For 20 patients we used germline DNA to filter for somatic variants and obtained 245 variants in total, while our model found seven variants, of which six were also detected using the germline strategy. In ten tumor-free individuals, our method detected in total one (potentially germline) variant, in contrast to 521 variants detected without our model. These results indicate that our model largely detects somatic variants.

## Introduction

With an increasing number of expensive treatments for breast cancer, there is a rising interest in predicting whether a specific treatment will be beneficial for a patient in a minimally invasive way. Circulating tumor DNA (ctDNA) could potentially play a role in this, but in ER + /HER2– Metastatic Breast Cancer (MBC) patients the clinical utility of ctDNA is currently limited. To date only mutations in the *PIK3CA* gene in ctDNA have direct clinical relevance, since they are predictive of response to alpelisib^1^. However next to upfront response prediction, dynamics in circulating tumor DNA (ctDNA) in the first weeks of treatment could potentially also be used to distinguish good and poor responders on the initiated treatment^2^. This would require an assay that is able to detect and quantify ctDNA in virtually all patients.

One way to detect and quantify ctDNA in the total pool of circulating cell-free DNA (cfDNA) is the detection of tumor-specific somatic variants^2^. Several targeted next generation sequencing (NGS) panels for breast cancer exist each covering different numbers of genes. Breast tumors in general are very heterogeneous and many different mutations in almost one hundred genes are involved in tumorigenesis^3–5^. Consequently, large targeted NGS panels preferably covering most if not all driver mutations are attractive since these are theoretically able to identify tumor-specific variants in a considerably higher fraction of breast cancer patients compared to smaller panels. On the other hand, any somatic variant detection method we use should have a low false discovery rate or equivalently high positive predictive value. This is because a false positive mutation can lead to mistakenly tracking either a sequencing error or a germline variant.

Furthermore, it is important to realize that large panels focusing on breast cancer genes differ from smaller panels not only in size of the genome which is sequenced, but also in the types of somatic variants that these panels target. Smaller panels include mainly oncogenes, in which one or a few well-known hotspot mutations that lead to activation of these genes are detected. In contrast, larger panels also include tumor suppressor genes, in which many different somatic variants could be the driver of inactivation. This renders the distinction between true tumor-specific somatic variants on one hand and sequencing artefacts and SNPs on the other hand more challenging.

Consequently, for optimal use and interpretation of sequence data of large NGS panels it is important to develop robust variant calling pipelines. For example, for the Qiaseq panels (Qiagen) no robust pipeline exists. As a result, one ends up with a large number of variant calls and it is not trivial to identify the true somatic variants among the sequencing artifacts and germline variants. In previous studies, several customized Qiaseq panels have been analyzed by in-house developed pipelines that often limited themselves to genes carrying hotspots^6–8^.

Here, we propose an approach to recognize somatic variants, in which the sequence characteristics and variant quality metrics of the well-known hotspot mutations are used to facilitate the identification of somatic variants in non-hotspot regions. For this purpose, we use sequencing data obtained in 70 ER + /HER2– MBC patients by both the Oncomine breast panel (Thermofisher) and the Qiaseq Human Breast Cancer Panel (Qiagen) to optimize the calling of somatic variants beyond the well-known hotspot regions by a machine learning algorithm using the well-established Oncomine panel^9–12^ as gold standard. We focused on Single Nucleotide Variants (SNVs) as they are by far the most common variants detected by Oncomine.

**Figure 1:**
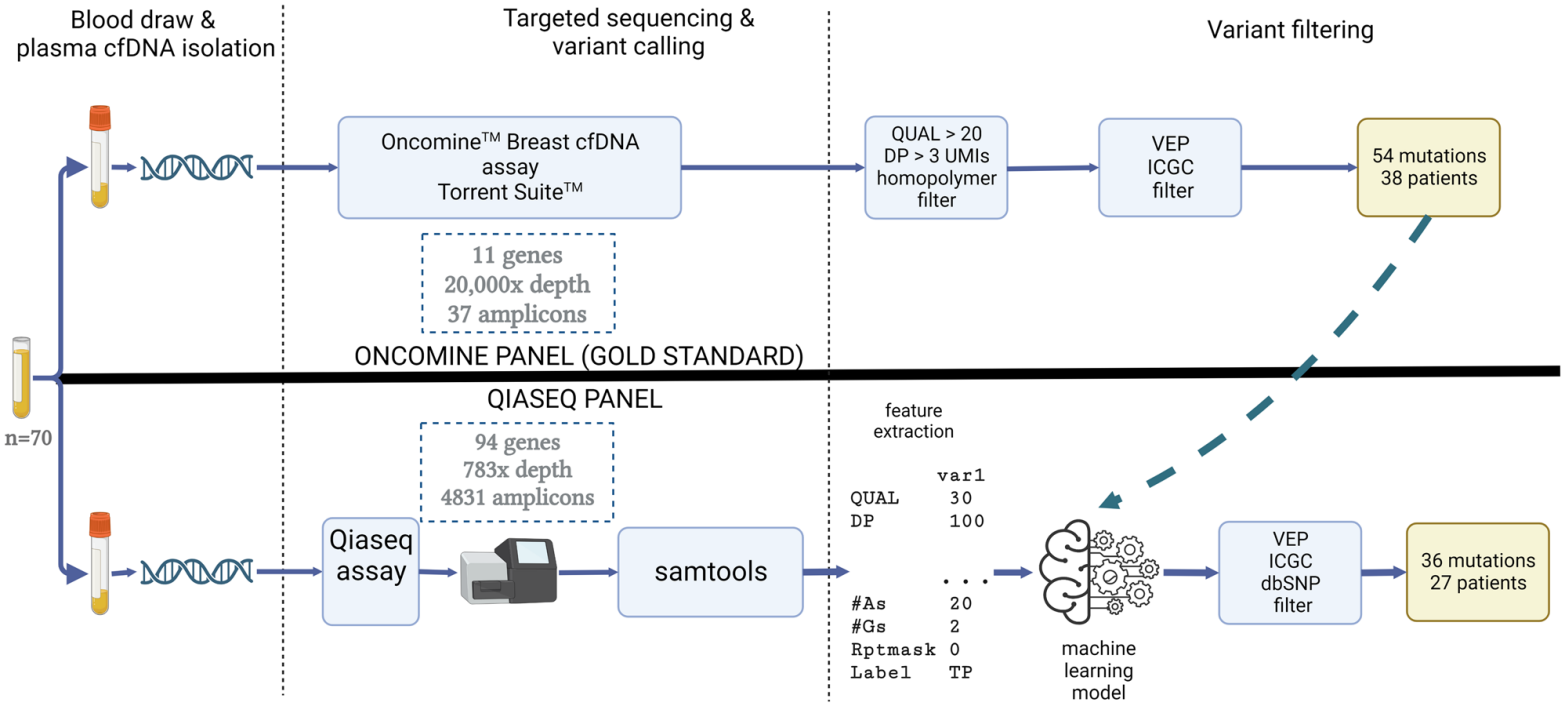
Overview of the experiments and comparison between the two mutation panels. Plasma cfDNA of 70 MBC patients was profiled using both the Oncomine (top) and Qiaseq (bottom) panels. We used the Oncomine calls in the regions shared by both panels to train a SVM classifier that predicts whether a variant called by Qiaseq would have also been called by Oncomine or not. Inputs to the model are features extracted from the variant caller output as well as from the sequence context. Then we applied post-processing filters on both panels to only keep variants that are most likely to be tumor-specific.

An overview of our approach is shown in Fig. 1. The code to reproduce our experiments and use our model is available at https://github.com/stamakro/largepanelsfiltering. Additionally, we compared the Oncomine breast panel (Thermofisher) and Qiaseq Human Breast Cancer Panel (Qiagen) by determining the fraction of patients with a detectable somatic mutation in their cfDNA for both panels and determining the concordance between both panels in ER + /HER2- metastatic breast cancer (MBC).

## Methods

### Blood processing and DNA extraction

Blood of 70 ER + /HER2– MBC patients was obtained immediately before the start of their first line of therapy. Blood was collected in Cell Save (Menarini) or BCT tubes (Streck) and processed to plasma within 96 h after collection. Cell-free DNA was extracted from 2 to 4 ml plasma using the Maxwell RSC ccfDNA Plasma Kit (Promega). Obtained cfDNA was quantified using the Qubit 2.0 dsDNA high sensitivity assay (Thermo Fisher Scientific).

### Ethical considerations

Informed consent was obtained from all participants. The study was approved by the accredited Medical Ehtics Committee of the Netherlands Cancer Institute-Antoni van Leeuwenhoek (METC AVL) and the study was performed in accordance with the Declaration of Helsinki.

### Library preparation and next generation sequencing

For both panels amplicon-based next generation sequencing libraries were prepared starting from 10 ng of cfDNA for all 70ER + /HER2– MBC patients according to the manufacturer’s instructions. A median 20.000 × read depth coverage for the Oncomine breast panel and a median 783 × read depth coverage for the Qiaseq Human Breast Cancer panel was obtained. These different read depths result in comparable costs for both panels.

### Oncomine variant calling and filtering

The Oncomine Breast cfDNA Assay v2 panel contains 37 amplicons covering about 150 hotspots, with a panel size of 1.9 kilobases (kb) and a median amplicon size of 48 bp [range 24–83 bp].

Base calling, read mapping and variant calling were performed using the Torrent Suite (BaseCaller, tmap and torrent variant caller, (Thermofisher)) with default settings. Only variants within the regions covered by the amplicons were called. We further focused on single nucleotide variants and removed variants if the “allele call” field was equal to “absent” or “no call” or if the PHRED quality score calculated by the torrent variant caller was below 20. Finally, we retained variants supported by at least 4 reads originating from unique molecules and removed one variant found in a homopolymer region (chr12:056492633, ERBB2, COSV57248104), as such regions are known to be challenging for Ion Torrent sequencing. We did not apply a minimum coverage filter, but all SNVs passing our filtering had coverage from 190 to 3054 unique molecules.

### Qiaseq variant calling and filtering

The Qiaseq panel contains 4831 amplicons, has a panel size of 565.2 kb and a median amplicon size of 117 bp [range 106–130].

After adapter trimming reads were mapped to the GRCH38 assembly using bwa–mem. Unique Molecule Identifiers (UMIs) were used to remove PCR duplicates and build a local consensus sequence. Then variants were called using bcftools mpileup, using the following settings: –Ov –regions 1 –annotate FORMAT/AD –min-MQ 10 –max-depth 10,000 –max-idepth 10,000 and bcftools call –Ov –multiallelic-caller –keep-alts. We only kept variants in chromosome 1–22 and the X chromosome that were in the panel’s target regions and also present in the COSMIC database (version 90). As we are interested in somatic variants, we removed homozygous calls (genotype 1/1 based on unique molecules) as well as sites with more than one variant alleles (genotype 1/2), because these are more likely to be sequencing errors. At this stage, we still had an unrealistic (high) number of COSMIC variants per sample, so we developed and compared two variant filtering strategies to remove false positive calls.

#### Rule-based filtering strategy

We only kept variants present in the COSMIC database and further applied filters on coverage, VAF (both estimated using unique molecules), and quality. For the coverage we tried 9 different values (10, 20, 50, 75, 100, 150, 200, 500 or 1000 unique molecules), for VAF 11 options (0.1%, 0.5%, 1.0%, 2%, 3%, 4%, 5%, 7.5%, 10%, 15%, 20%), and for quality 10 options (1, 2, 5, 10, 15, 20, 25, 30, 40, 50). Trying all possible combinations gives 990 different filtering strategies.

#### SVM model to filter variants

We trained a model to predict whether a given variant identified using the Qiaseq panel is also called by Oncomine in the same patient. We focused on the regions that are covered by both panels (Supplementary Table 1) and for this experiment we treated the Oncomine variants as ground-truth, i.e. assumed that any variants missed by Qiaseq in those regions are false negatives and any variants found by Qiaseq but not by Oncomine are false positives. In that case, variant filtering becomes a binary classification task (“called by Oncomine” vs “not called by Oncomine”). We extracted two types of features from each Qiaseq call: *type* (1) Sixteen properties of the variant call based on the output of mpilup: quality, segregation-based metric, p-values for read position bias, mapping quality bias, mapping quality vs strand bias, and base quality bias, allele count, the number of high quality bases supporting the reference and the variant allele in each strand (2 × 2 features), mapping quality, the number of reference alleles in the called genotype (2 for ‘0/0’ and 1 for ‘0/1’ genotype calls’) coverage, VAF and the number of unique molecules supporting the variant; and *type *(2) Six properties of the sequence around the location of the variant in a 60 bp window (30 bp upstream and 30 bp downstream): the number of occurrences of A, C, G and T, the GC fraction and the fraction of bases masked as repetitive by RepeatMasker (OSS:6.5.7.0). Repeat characterization was performed using RepeatMasker (Smit, AFA, Hubley, R & Green, P. RepeatMasker Open-4.0. 2013–2015; http://www.repeatmasker.org) in a 60 bp window centered around the variant. The 22-dimensional vectors of each variant call were standardized to zero mean and unit variance and fed into a support vector machine (SVM) classifier. We tried both a linear and a RBF (Radial Basis Function) kernel. For the C parameter that controls the regularization as well as for the precision parameter of the RBF kernel (γ) we tried the values (1e–4, 1e–3, 1e–2, 0.1, 0.5, 1, 2, 5, 10, 20, 50).

### Tuning and evaluation of Qiaseq filters

To be able to evaluate our different filtering approaches on independent unseen data, we performed a leave-one-patient-out experiment. At each iteration, the Qiaseq calls in the regions covered by both panels from one of the 70 patients constitute our test set and the calls from the remaining 69 patients our training set. Within our training set we performed stratified fourfold cross-validation (pooling all variants from the 69 patients) in order to tune the parameters of our models (for our rule-based filtering strategy, the optimal VAF, coverage and quality cut-offs; for the linear SVM the C parameter and for the kernel SVM the C and γ parameters) using as criterion the F1 score.

The parameter combination that maximized the mean value of the criterion across the 4 folds was selected. For our rule-based filtering strategy, this meant that the optimal cut-offs were applied to the Qiaseq calls of the test patient. For our model-based filtering, the best-performing kernel function, C value and γ (if applicable) were used to train a new model on all variant calls from the entire training set which was then applied to the test patient. We evaluated the performance on the Qiaseq calls of each test patient in terms of precision (*pr*, one minus the false discovery rate), recall (*rc*, also known as sensitivity), F1 score, Matthews correlation coefficient (MCC), and Youden’s J. These are defined in Eqs. (1), (2), (3), (4) and (5), where TP, TN, FP and FN denote the number of true positives, true negatives, false positives and false negatives respectively.

1 $$pr=\frac{TP}{TP+FP},$$

2 $$rc=\frac{TP}{TP+FN},$$

3 $$F1=\frac{2\cdot pr\cdot rc}{pr+rc},$$

4 $$MCC= \frac{TP\cdot TN-FP\cdot FN}{\sqrt{(TP+FP)(TP+FN)(TN+FP)(TN+FN)}} ,$$

5 $$J=\frac{TP}{TP+FN}+\frac{TN}{TN+FP}-1.$$

To use our model on regions not covered by Oncomine and to new patients, we repeated the fourfold cross-validation procedure on the data from all 70 patients, selected the best performing parameters and re-trained the model on all the data with those parameters.

### Removing synonymous mutations and germline variants

We additionally performed some post-processing filters to remove likely germline or benign variants and to keep only somatic variants with high confidence. The variants that passed the filtering were fed to the Ensembl Variant Effect Predictor (VEP)^13^ to assess their effects on the transcript, selecting the most severe of all possible consequences for each variant. We kept only variants with one of the following consequences: (missense_variant, start_lost, stop_lost, stop_gained, splice_acceptor_variant, splice_donor_variant, splice_donor_5th_base_variant). Subsequently, to remove likely germline variants, only variants which were detected in at least one patient in TCGA (n = 486), Nik Zainal et al.^3^ (n = 324) or in the whole genome sequencing results of 72 breast cancer patients obtained from the ICGC data portal (BRCA-FR), were considered to be somatic. Only patients with ER + /HER2– breast cancer from the TCGA cohort were included since this cohort matches our in-house cohort (ER + /HER2– MBC). Variants which were not present in any of these datasets were removed.

Lastly, variants whose VAF was indicative of a heterozygous or homozygous germline variant that were present in dbSNP^14^ were removed. Hereto, we used the VAFs of all variants passing the model filtering (16,717 variants) in the Qiaseq-specific regions of the 70 patients to fit a mixture of four beta distributions. We expect that these variants include sequencing errors, true somatic variants, germline heterozygous and germline homozygous variants. Therefore, each of the four components was initialized to bias the model towards these four categories (Fig. S1). After fitting the mixture model to our data, we can use each mutation’s VAF to assign it to the most likely component. Based on the results of the fitting procedure, for the germline heterozygous variants, that corresponds to a VAF in the range [36–67%] and for germline homozygous to the range [67–100%].

These post-processing filters were applied to both Oncomine and Qiaseq variant calls. However, for the comparison of our model to rule-based filtering and to no filtering in the leave-one-patient-out scheme (see above), we omitted these post-processing filters for both panels. This was done so that we can evaluate the variant filtering ability of each method irrespective of the effects of the variants. Additionally, we assessed whether our model has additional value compared to only applying the post-processing filters. To this end, we evaluated the filtering strategy of keeping all variant calls that pass the post-processing filters without considering any other information about the call other than chromosome, position and alternate allele. We compared this approach to our model in the leave-one-patient-out setting.

### Additional validation experiments

To investigate the reliability of the identified variants in the regions unique to the Qiaseq panel, we used data from 8 patients showing variants in both panels. We fitted a linear regression on the logits of the VAFs using the mutations called by both panels (24 mutations) predicting the Qiaseq VAF from the Oncomine VAF. More specifically, we employed a Bayesian linear regression without an intercept. As we expect the Oncomine and Qiaseq VAFs to be positively correlated and almost equal we used a N(1, 0.5) prior for the slope. For the variance of the error, we used an exponential prior with mean of 1 and the model was fit using Markov Chain Monte Carlo sampling.

To further test the generalization ability of the model on new data and assess our approach’s false discovery rate, we employed it on three additional Qiaseq datasets:

The first included 69 additional MBC patients without an Oncomine mutation, so we expect to find no mutations in the shared regions between the panels, but potentially some variants are present in the Qiaseq-specific regions. Second, we profiled 9 healthy blood donors, for whom we do not expect any tumor-derived somatic mutations at all. Third, germline DNA isolated from peripheral blood mononuclear cells (PBMCs) of 20 patients in our original dataset of 70, in which we do not expect to detect tumor-specific mutations.

The library preparation, sequencing and processing of these samples were carried out as described above, but in different batches and different sequencing depths. Specifically, the median read depth for the 69 MBC patients was 528 x, for the 9 HBDs 1557 x, and for the 20 germline samples 3820 x.

Additionally, we used the (unfiltered) variant calls of these 20 germline samples to call somatic variants in the corresponding cfDNA samples. In this experiment, any variant called in a cfDNA sample but not in the corresponding germline sample was labelled somatic and subsequently fed to our post-processing filters. We then compared this approach to our machine learning-based filter.

## Results

### Oncomine panel identifies mutations in 38 of the 70 patients

cfDNA of seventy patients were analyzed with the Oncomine assay, without analyzing the germline of these patients. In total 38 samples (54%) had at least one somatic mutation detected. On average, we found 1.4 variants [0–4] per patient. These 38 ctDNA-positive samples included seven samples (10%) with two variants and four samples (6%) with three or more variants. In total, 54 variants were detected in 7 genes (*PIK3CA, TP53, ESR1, AKT1, ERBB2, ERBB3, KRAS*). The specific mutations detected in each patient by the Oncomine panel are shown in Table S2. The highest number of mutations, in total 37, were found in the *PIK3CA* gene, while in total 10 mutations were detected in the *TP53* gene and 2 mutations in both the *ESR1* and the *AKT1* gene. As for occurrence in each patient, in total 30/70 (42.9%) patients had a mutation detected in *PIK3CA*, 9/70 (12.9%) patients had a mutation detected in *TP53*, 2/70 (2.9%) patients had a mutation detected in *AKT1* and 1/70 (1.4%) patients had two mutations detected in *ESR1*. The VAFs of the mutations ranged from 0.2 to 53.2% with a median of 3.4%. In total 10 of the 54 (18.5%) mutations had a VAF below 1.0% (Table S2).

### Large false positive rate of the Qiaseq panel alleviated by machine learning-based filtering

A larger panel could potentially increase the subset of patients in which at least one mutation is detected (54% with the smaller Oncomine panel). Therefore, cfDNA from the same blood samples was also sequenced with the Qiaseq panel which includes all regions of the Oncomine panel except two regions: one in the SF3B1 gene and one in the FBXW7 gene. This results in 8 genes from which the Oncomine and Qiaseq panel have regions in common. Next to these overlapping regions (Table S1) the Qiaseq panel covers 4796 additional regions in 86 additional genes.

**Figure 2:**
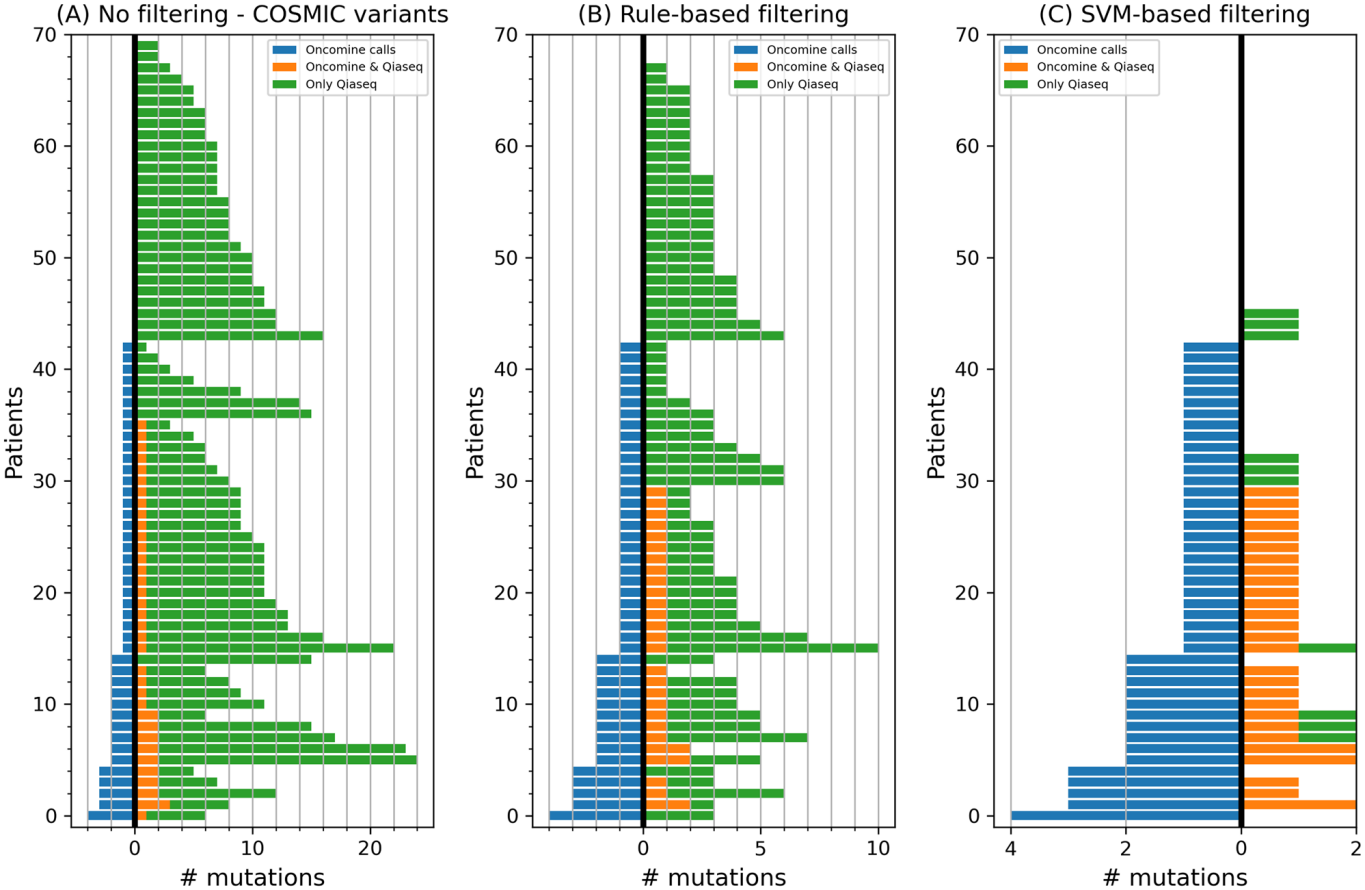
Number of COSMIC variants (*x-*axis) detected by Oncomine and Qiaseq in the common regions between the two panels when we applied (**A**) no filtering, (**B**) our rule-based filtering strategy, and (**C**) our proposed SVM learning model to filter somatic mutations. We show the results for each of the 70 patients (*y-*axis) when we used the remaining 69 patients to tune our filters. In blue, on the left part of the *x-*axis, we show the number of Oncomine mutations for each patient. The number of those mutations also detected by Qiaseq is shown on the right as orange bars. Green bars denote the number of additional mutations found by the Qiaseq panel for that sample. The number of variants which are shown, are without applying the post-processing filters.

From the Qiaseq data, we obtained on average 23.4 variants [4–55] per patient in the regions common to the regions targeted by the Oncomine panel (common regions). However, based on data from the International Cancer Genome Consortium, we expect that breast cancer patients have on average 1.9 somatic mutations in the entire coding sequence of these 8 genes (genes with common regions), suggesting many of these variants are false positives. If we restrict to only variants present in the COSMIC database, we still obtained a high number of likely false positives (9.1 [1–24] variants per patient on average in the common regions) (Fig. 2A).

To reduce the number of false positive variants obtained with the Qiaseq panel, we considered the Oncomine calls in the common regions as ground truth and used them to optimize the variant filtering strategy for the Qiaseq data. Qiaseq variants were either filtered based on VAF, coverage and quality (“rule-based filter”; Fig. 2B), or a machine learning model (SVM model) that uses several quality metrics of each variant as well as the sequence context (Fig. 2C). Table 1 shows the Qiaseq results compared to the Oncomine calls for the regions in common between the 2 panels for the different filtering approaches. Precision-recall curves of our SVM model using fourfold cross validation are shown in Fig. S2. Note that for this comparison, we did not employ the post-processing filters (VEP, ICGC and dbSNP). Our SVM model outperformed the rule-based filtering on all performance metrics we evaluated (precision, recall, F1 score, Matthews correlation coefficient (MCC), Youden’s J). In addition, the MCC and Youden’s J values are zero, i.e. equal to the performance of random guessing, when no filtering technique is used, highlighting the necessity of our method.

**Table 1:** Evaluation of different filtering strategies for Qiaseq variant calls using the Oncomine calls in the common regions as ground truth.

	Precision	Recall	F1 score	MCC	Youden’s J
No filtering	0.07	1	0.12	0	0
Rule-based filtering	0.12	0.33	0.17	0.17	0.19
SVM-based filtering	0.36	0.35	0.35	0.34	0.33
Post-processing filtering only	0.20	0.4	0.26	0.23	0.22

*MCC* Matthews correlation coefficient, *SVM* support vector machine.

As in subsequent analyses we apply additional filters on the variants selected by the model, we tested whether the model still has added value or is overshadowed by the post-processing filtering. We found that the post-processing filters alone failed to match the performance of our model in the leave-one-patient-out experiment (Table 1). Specifically, this filtering strategy found more true Oncomine variants than our model (recall of 0.4 vs 0.35), but at the cost of also calling considerably more false positives (precision of 0.2 vs 0.4), leading to worse overall performance in terms of F1 score, MCC, and Youden’s J. Interestingly, the post-processing filters did outperform the rule-based filters.

Collectively, these results show that simple filtering rules such as VAF and depth or selecting for well-known variants are not sufficient for optimal filtering and that our model managed to learn subtler patterns leading to an overall increased performance.

### Oncomine and Qiaseq panels have comparable results in variants and variant allele frequency when sequencing at the same depth

When we applied our model to data from the unique regions of the Qiaseq panel, 11 variants were detected in 10 out of the 70 evaluated patients (Table S2). These variants consist of 4 mutations in CDH1, 1 mutation in KMT2C, 2 mutations in TP53, 1 mutation in SMARCA4, 1 mutation in PTEN and 1 mutation in CBFB. Using data from 8 patients that carried variants in both the unique and common regions, observed VAFs were within the expected range for the majority of variants in the unique regions, indicating the validity of these additional mutations (Fig. S3).

When we focused on the common regions between both panels, the Qiaseq panel only detected 24 out of the 54 variants (44.4%) which were detected by Oncomine. In total 30 variants were not detected by Qiaseq. In addition, one variant not detected by Oncomine (PIK3CA p.P539R, COSV55876380) was detected by Qiaseq and passed all our filters in one patient. Mutations not detected by the Qiaseq panel had a lower median variant allele frequency (VAF) as determined by the Oncomine panel compared to those that were detected by the Qiaseq panel (1.8% (IQR: 0.9–3.2%) vs. 14.4% (IQR 7.3–29.0%), p value ≤ 0.0000001, Mann–Whitney test), suggesting that the difference in sequencing depth of both panels is the main cause for the limited number of mutations Qiaseq detects.

**Figure 3:**
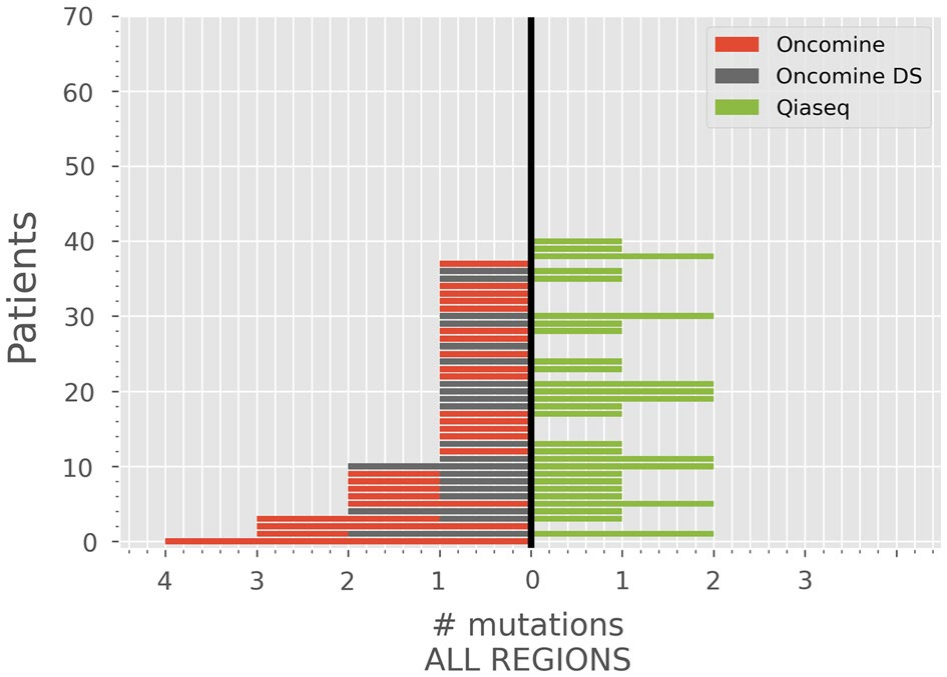
Number of mutations (*x*-axis) detected per patient (*y*-axis) using Oncomine (red), down-sampled Oncomine (gray-black), and Qiaseq (green). All regions covered by each panel are included.

To further investigate the effect of this difference in coverage, we down-sampled the Oncomine sequencing reads in silico to a coverage level comparable with the coverage of the corresponding patient sample in the Qiaseq panel and repeated the variant calling (Fig. 3). After down-sampling, the number of variants detected by Oncomine decreased from 54 variants in 38 patients to 23 variants in 20 out of the 70 patients. Twenty of these 23 mutations were detected by the Qiaseq panel. Mutations which were not detected in the down-sampled samples had a lower median VAF (8.6% vs. 21.6% for the detected variants, p value ≤ 0.000001, Mann–Whitney test) in the full-coverage samples. In total 20 of the 25 detected mutations in the common regions with the Qiaseq panel were detected with the Oncomine panel when down-sampled.

In addition, we observed that the VAF estimations obtained by the three approaches (Oncomine, downsampled Oncomine, Qiaseq plus SVM filtering) were highly concordant (Fig. S4). The VAFs of the 24 mutations detected by both Oncomine and Qiaseq had a Spearman correlation of 0.92 and a Median Absolute Deviation (MAD) of 3.83%. For the VAFs obtained by the full and down-sampled Oncomine assays (n = 23) we found a MAD of 1.15% and ρ = 0.91 and finally between Qiaseq and down-sampled Oncomine (n = 19) a MAD of 3.60% and ρ = 0.87. These results show that the Qiaseq panel combined with our post-processing filters can reliably detect variants and accurately estimate VAFs if the coverage is sufficiently large.

### Validation of the model in independent MBC patients in which no mutation was detected by the Oncomine panel

Subsequently, our machine learning model was applied to sequencing results of a different set of cfDNA data of 69 MBC patients in which no somatic variants were detected by the Oncomine panel. Sequencing with both panels was performed methodologically as described in the methods. In those patients, the Qiaseq panel detected a median of 535 COSMIC heterozygous SNVs (IQR = [388–697]). 67 of these patients had at least one such variant called in the regions that were also assessable by Oncomine, with a median of 3.5 variants and a maximum of 20. After feeding these variants to our machine learning model and applying the post-processing filters, all of the variants called by Qiaseq in the regions shared by Oncomine were filtered out.

In the remaining regions we found three additional variants in three patients: two KMT2C mutations and one PIK3CA mutation. These results further corroborate the observation that our model and post-processing filters can match the output of the Oncomine panel, generalizing its application to completely unseen samples sequenced in a different batch.

### cfDNA data from 9 healthy donors demonstrate the utility and precision of the model

To further evaluate the performance of our model for filtering out false positive calls, we profiled cfDNA from 9 healthy individuals, who are not expected to have any tumor-specific somatic mutations, using the Qiaseq panel. Out of the 331,349 called variants, we found a total of 18,117 COSMIC SNVs. We fed these variants into the model, which flagged about 89% of them as false positives. From the remaining 1997 variants, about 84% were present in dbSNP, which implies that most of the variants that are selected by the model are likely to be real germline variants. Indeed, our dbSNP filter removed 55% of the variants leaving us with 897 calls. To restrict ourselves to possible somatic, tumor-specific mutations with high confidence, we further selected the variants based on their effect on the transcript and their presence in previous breast cancer studies (see “Methods”), leaving us with 1 variant (COSV54683559, TGFB1 gene).

This last step will likely also remove potential variants originating from clonal hematopoiesis (CH), which is essential since a previous study showed that the prevalence of CH variants is high in cfDNA of healthy individuals^15^. Indeed, the ICGC filter is the most stringent one, as applying it by itself gave only two variants, while 377 variants passed both the VEP and dbSNP filters. The second variant found by only applying the ICGC filter was most likely a germline variant, because it had a VAF of 46.6% at 283 × coverage and was a known variant in dbSNP (rs201792218). This variant was removed by our dbSNP filter.

These results show that in only one of the 9 healthy donors, we find one known somatic mutation (COSV54683559, TGFB1 gene) at a VAF of 27.4%. This variant was not reported as a potential CH variant by Razavi et al.^15^ who sequenced tumors, plasma and WBCs at a high sequencing depth.

Bypassing our newly developed model and directly applying the VEP, ICGC and dbSNP filters on all COSMIC SNVs detected leaves us with a total of 378 variants in the 9 healthy donors. Only 1 of those variants (0.2%) is in dbSNP (but not in the germline VAF range), meaning that in this way we mainly keep true somatic variants and/or sequencing errors. The median VAF of these 378 mutations is 0.32% (IQR [0.21–0.51%]), which, given the low sequencing depth, points to many of them being actually errors. 31 out of the 378 mutations (8%) had a VAF larger than 1% and could be considered as somatic variants, meaning that on average we find 3.4 tumor-specific variants per healthy blood donor. As we do not expect this many mutations in these healthy subjects, this experiment demonstrates the practical utility of our machine-learning-based filtering in conjunction with the post-processing steps.

### Model-based filtering is also applicable to germline DNA

To evaluate the model’s potential to identify true somatic variants we sequenced germline DNA with the Qiaseq panel from peripheral blood mononuclear cells of 20 MBC patients for which cfDNA Qiaseq data was also available. Sequencing these germline samples resulted in 151,520 variants after basic filtering. To test whether our machine learning model can also be applied to sequencing results from germline DNA, we applied the model to these 151,520 variants which resulted in 4358 variants. In total 65% of these variants are likely germline variants based on their VAF and the fact that they are known in dbSNP. After applying the additional filters all variant calls were filtered out. This consistent with our expectation that there shouldn’t be any tumor-specific variants in germline DNA.

Notably, these 20 samples were sequenced at 5 to 10 times higher depth than the cfDNA samples used for building the model. Despite this difference, the model selected a similar average number of variants per patient as previously, implying that use of the model can also be generalized to different sequencing depths than the training data.

### Model-based filtering of Qiaseq variant calls as a possible alternative to germline sequencing

Subsequently we used the matched germline DNA as a filter to remove germline variants from the sequencing results of the cfDNA samples by filtering out all cfDNA variants that were also present in the matched germline sample. This approach resulted in 10,801 remaining variants. After sequentially applying the VEP, dbSNP and ICGC filters 8014, 7906 and 245 variants remained respectively in the 20 patients. Again, the ICGC filter removed the most variants, but the other two filters, VEP and dbSNP, removed 78 different variant calls. This shows that it can be beneficial to use all of our proposed post-processing filters if the goal is to minimize false positives.

Alternatively, applying our model to these 20 patients resulted in 4693 variants, i.e. less than half of the amount that passed the matched germline filter. Of those variants, seven remained after the ICGC filter, the VEP filter and the dbSNP filter. Six of these seven also passed the germline DNA filter, indicating they were not present in the germline DNA from these patients and can therefore be interpreted as true somatic variants. The one variant that did not pass the germline filtering, was present in the matched germline sample with a VAF of 1.5% and is therefore unlikely to be a germline mutation. Interestingly, the VAF for this mutation in the accompanying cfDNA was considerably higher (19.1%), suggesting the germline sample may have been inadvertently contaminated with a low amount of cfDNA. These results indicate that our model is able to identify high-confidence somatic variants. On the other hand, we cannot exclude that the model eliminates additional bona fide somatic variants, since filtering by germline variants leaves many more potential somatic variants than the model does. It is unlikely that all these variants are true somatic variants since the median VAF of these variants is 0.8% (IQR 0.5–1.7%) compared to a median VAF of 11.5%, of the seven variants detected by the model (Fig. S5).

### Mutational landscape of first line MBC ER + patients

When we merge the Oncomine and Qiaseq sequencing results together, at least one mutation was detected in cfDNA of 41 (58.6%) of the 70 patients. In supplemental Table S2 the specific mutations which were detected by the Oncomine and/or Qiaseq panel are shown.

**Figure 4:**
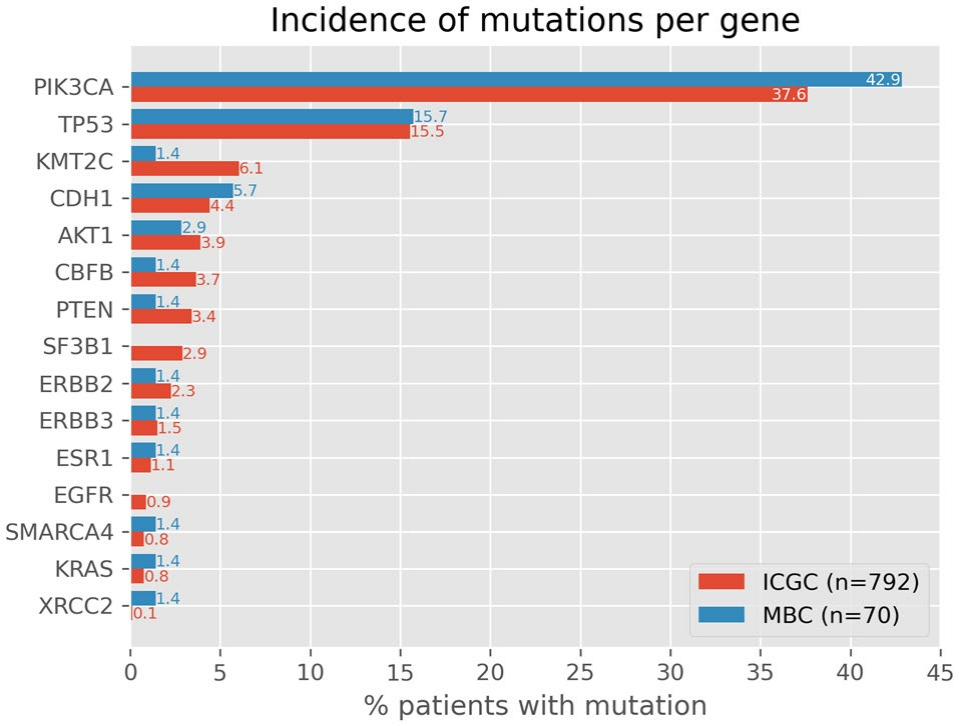
Incidence of mutations per gene detected in our cohort by the Qiaseq and Oncomine panels compared to the incidence of mutations per gene in the TCGA and BASIS cohorts.

To validate our results, we compared the incidence of the detected variants in this study with those in two tissue datasets of patients with primary breast cancer: The Cancer Genome Atlas (TCGA)^16^ and BASIS cohorts^17^ obtained from the International Cancer Genome Consortium (ICGC)^18^. Figure 4 shows that the incidences of mutations in the TCGA and BASIS cohorts were comparable with our cohort consisting of patients with metastatic breast cancer (Spearman ρ = 0, 64, p-value = 0.0098), before start of first line treatment.

## Discussion

We have developed a machine-learned model to reliably identify tumor-specific somatic mutations from the large Qiaseq breast NGS panel without the need for germline sequencing data or a panel of normal control samples. The machine learning model was developed based on sequencing results of cfDNA of metastatic breast cancer patients and subsequently also applied to cfDNA of an independent cohort of comparable patients. The model was first trained using sequencing characteristics obtained for high confidence hotspot mutations obtained by the smaller Oncomine breast panel. The results of applying the model on sequencing results of the large NGS panel in these patients were subsequently verified by evaluating NGS data from cfDNA from healthy blood donors and germline DNA of part of the included subjects. The consistency of the model was demonstrated by the equal number of variants passing the filters applied by the model on NGS data of germline DNA from WBCs and of cfDNA from HBDs. In addition, results for the overlapping hotspot mutations between both panels were highly correlated in the cases where VAF and sequencing depth allowed the detection of the mutations by both panels.

In the development of our machine learning model and subsequent additional filtering steps, we balanced the importance of detecting all potential somatic variants (sensitivity) and the confidence of the detected variants to be tumor-specific (specificity). Our focus was on calling tumor-specific somatic mutations with high confidence. For this reason, after applying the model, we further selected for those variants that were highly pathogenic and which had previously been detected in at least one of three large breast cancer tissue datasets. However, while focusing on confidence, it is inevitable that part of the true somatic variants will be missed. Since we recognize that the balance between specificity and sensitivity is very dependent on the research and/or clinical question, we designed our model in such a way that these additional filtering steps can be omitted or adapted when desired, for example by including other or additional variant databases. In addition, the SVM classifier outputs a score for each variant (also known as decision function) with positive scores indicating that the variant is more likely to be true than a false positive. We set the threshold for calling a variant at zero, but this can also be increased or decreased if a specific application requires higher specificity or sensitivity, respectively.

One limitation of our work is that we focused on single nucleotide substitutions and did not consider other variants. However, we expect that deactivation of tumor suppressor genes is likely to be triggered by large deletions or small frameshift insertions and deletions than only point mutations, while that is not usually the case for oncogenes. That means that calling indels reliably (to the extent that the short reads allow it) is necessary to maximize the potential of employing large panels in clinical practice. Extracting variant calling and sequence context features for indels can be done similarly to our work here but other features, such as the size of the aberration, are also likely to be informative. However, the Oncomine panel detected only a handful of small indels in our set of 70 patients, a large fraction of which were in homopolymer regions which makes them less reliable than the SNV calls in the same panel. Homopolymer regions are known to be challenging for Ion Torrent sequencing^19,20^. In the absence of a reliable gold standard, it is hard to train a supervised learning model as the one we built for SNVs. Future work could focus on creating such a standard by profiling cfDNA and matched tumor samples with the same panel and treating indels called in both as true positive examples and indels called only on cfDNA as negative examples. A set of healthy individuals might also be utilized to flag regions where indels are often called due to biases of the assay. Purely data-driven approaches will be more challenging, as there is no set of well-known hotspot indels in these tumor suppressor genes to enable a supervised or positive-unlabeled learning method^21^, while that would have been possible with the oncogenes.

Finally, our model is trained to predict whether the Oncomine panel would have called a specific variant based on the variant call features and it is very likely that the Oncomine would have also called germline variants with a VAF around 50%. However, the oncogenic regions that are assessable by both panels considered in this study do not contain common germline variants. That makes the distinction between somatic and germline variants challenging for our classifier and any other data-driven filtering approach, such as putting a minimum VAF threshold. The post-processing steps we used help enrich our variant calls for somatic mutations without the need of matched germline DNA sequencing or a panel of unmatched normal samples which reduces the cost of the assay, making it more suitable for clinical practice. As in the case of indels above, the generation of matched tumor-normal data from these panels will help create a ground truth dataset that can be used to distinguish somatic from germline variants.

In practice, the choice for the right panel is also influenced by the associated costs. Results from the current study, in which cfDNA from 70 MBC patients was analyzed by two NGS panels, show that a small hotspot panel at a high sequencing depth is able to detect ctDNA in more patients than a large panel at a lower sequencing depth to keep costs equal. However, when sequenced at a similar depth, there is added value for the use of a larger panel. Profiling patients with the Qiaseq panel at a higher depth will increase the cost, but will also result in detecting variants in a larger subset of patients.

## Conclusion

We have developed a machine-learned model to reliably identify tumor-specific somatic mutations from the large Qiaseq breast NGS panel without the need for germline sequencing data. Our model takes advantage of the sequencing characteristics of well-known hotspot mutations to subsequently call somatic variants in tumor suppressor regions without clear hotspots. The model can generalize to Qiaseq data sequenced at larger depths than the training set and to genomic DNA. Our analyses underlined that our model was able to identify true tumor-specific somatic variants with high confidence. We expect a similar approach will be beneficial for the analysis of other NGS panels as well. Additional filtering steps are provided separately from the model to allow future users to fine-tune these to their specific needs.

## Supplementary Information

Supplementary Information.

## Data Availability

The datasets used and/or analyzed during the current study are available from the corresponding author on reasonable request.
