# Supplementary material for: Machine learning-based somatic variant calling in cell-free DNA of metastatic breast cancer patients using large NGS panels
Source: Sci Rep. 2023 Jun 27;13:10424. doi: 10.1038/s41598-023-37409-1 (PMC10300101; doi:10.1038/s41598-023-37409-1)
Supplement: Supplementary file 1 — Supplementary Information. [file 41598_2023_37409_MOESM1_ESM.docx]

**Supplemental data**

| **Table S1** Common regions between two panels (ghrch38) |
| --- |
| \| chr3 \| 179203735 \| 179203784 \| PIK3CA \| \| --- \| --- \| --- \| --- \| \| chr3 \| 179210162 \| 179210198 \| PIK3CA \| \| chr3 \| 179210278 \| 179210302 \| PIK3CA \| \| chr3 \| 179218278 \| 179218320 \| PIK3CA \| \| chr3 \| 179221106 \| 179221154 \| PIK3CA \| \| chr3 \| 179234277 \| 179234314 \| PIK3CA \| \| chr6 \| 152011685 \| 152011744 \| ESR1 \| \| chr6 \| 152094382 \| 152094421 \| ESR1 \| \| chr6 \| 152098779 \| 152098822 \| ESR1 \| \| chr7 \| 55191750 \| 55191833 \| EGFR \| \| chr12 \| 25245337 \| 25245376 \| KRAS \| \| chr12 \| 56085039 \| 56085087 \| ERBB3 \| \| chr12 \| 56088129 \| 56088171 \| ERBB3 \| \| chr12 \| 56088530 \| 56088578 \| ERBB3 \| \| chr12 \| 56088808 \| 56088860 \| ERBB3 \| \| chr12 \| 56098838 \| 56098900 \| ERBB3 \| \| chr14 \| 104780185 \| 104780228 \| AKT1 \| \| chr17 \| 7669648 \| 7669693 \| TP53 \| \| chr17 \| 7670567 \| 7670643 \| TP53 \| \| chr17 \| 7670692 \| 7670727 \| TP53 \| \| chr17 \| 7673532 \| 7673609 \| TP53 \| \| chr17 \| 7673686 \| 7673722 \| TP53 \| \| chr17 \| 7673760 \| 7673839 \| TP53 \| \| chr17 \| 7674187 \| 7674264 \| TP53 \| \| chr17 \| 7674863 \| 7674896 \| TP53 \| \| chr17 \| 7674940 \| 7674972 \| TP53 \| \| chr17 \| 7675061 \| 7675099 \| TP53 \| \| chr17 \| 7675138 \| 7675219 \| TP53 \| \| chr17 \| 7675962 \| 7676008 \| TP53 \| \| chr17 \| 7676054 \| 7676127 \| TP53 \| \| chr17 \| 7676174 \| 7676208 \| TP53 \| \| chr17 \| 7676253 \| 7676297 \| TP53 \| \| chr17 \| 7676376 \| 7676419 \| TP53 \| \| chr17 \| 7676435 \| 7676440 \| TP53 \| \| chr17 \| 7676545 \| 7676612 \| TP53 \| \| chr17 \| 39723961 \| 39723993 \| ERBB2 \| |

**Table S2**: Overview of the detected variants and VAF by the Oncomine and the Qiaseq panel

|  |  |  | **Oncomine panel** | | | **Qiaseq panel** | | | |
| --- | --- | --- | --- | --- | --- | --- | --- | --- | --- |
| **Subject** | **Gene** | **Variant** | **Molecular coverage** | **Alt reads** | **VAF** | **Molecular coverage** | **Alt reads** | **VAF** | **Called** |
| 1 |  |  |  |  |  |  |  |  |  |
| 2 | PIK3CA | p.E545K | 1158 | 21 | 1.8 | 294 | 4 | 1.4 | 0 |
|  | PIK3CA | p.G1049R | 1794 | 47 | 2.6 | 128 | 2 | 1.6 | 0 |
|  | TP53 | p.R280K | 736 | 7 | 1 | 110 | 2 | 1.8 | 0 |
|  | TP53 | p.P250L | 1135 | 5 | 0.4 | 89 | 0 | 0 | 0 |
| 3 |  |  |  |  |  |  |  |  |  |
| 4 | PIK3CA | p.E545K | 1359 | 668 | 49.2 | 538 | 243 | 45.2 | 1 |
|  | CDH1 | p.Q23* | not covered by panel | | | 42 | 11 | 26.2 | 1 |
| 5 | PIK3CA | p.H1047R | 1725 | 4 | 0.2 | 187 | 3 | 1.6 | 0 |
| 6 | PTEN | p.D92E | not covered by panel | | | 283 | 53 | 18.7 | 1 |
|  | CDH1 | c.1565+1G>A | not covered by panel | | | 82 | 14 | 17.1 | 1 |
| 7 |  |  |  |  |  |  |  |  |  |
| 8 | PIK3CA | p.H1047R | 1451 | 4 | 0.3 | 67 | 0 | 0 | 0 |
| 9 | AKT1 | p.E17K | 558 | 198 | 35.5 | 87 | 28 | 32.2 | 1 |
|  | CBFB | p.Q67H | not covered by panel | | | 230 | 60 | 26.1 | 1 |
| 10 |  |  |  |  |  |  |  |  |  |
| 11 |  |  |  |  |  |  |  |  |  |
| 12 | PIK3CA | p.E545K | 1216 | 322 | 26.5 | 343 | 85 | 24.8 | 1 |
| 13 | PIK3CA | p.E453K | 657 | 91 | 13.85 | 680 | 44 | 6.5 | 1 |
|  | PIK3CA | p.H1047R | 1048 | 93 | 8.9 | 192 | 33 | 17.2 | 1 |
| 14 | PIK3CA | p.H1047R | 624 | 53 | 8.5 | 106 | 4 | 3.8 | 0 |
| 15 |  |  |  |  |  |  |  |  |  |
| 16 |  |  |  |  |  |  |  |  |  |
| 17 |  |  |  |  |  |  |  |  |  |
| 18 | PIK3CA | p.H1047R | 2543 | 379 | 14.9 | 167 | 31 | 18.6 | 1 |
|  | PIK3CA | p.P539R | not covered by panel | | | 357 | 41 | 11.5 | 1 |
|  | KRAS | p.G12V | 2922 | 32 | 1.1 | 174 | 0 | 0 | 0 |
| 19 | PIK3CA | p.H1047R | 1078 | 11 | 1 | 285 | 9 | 3.2 | 0 |
| 20 | KMT2C | p.K822R | not covered by panel | | | 713 | 9 | 1.3 | 1 |
| 21 |  |  |  |  |  |  |  |  |  |
| 22 | PIK3CA | p.E542K | 801 | 8 | 1 | 555 | 18 | 3.2 | 1 |
| 23 | KMT2C | p.H367Y | not covered by panel | | | 468 | 7 | 1.5 | 1 |
|  | TP53 | p.K132R | 1045 | 13 | 1.2 | 45 | 0 | 0 | 0 |
| 24 |  |  |  |  |  |  |  |  |  |
| 25 |  |  |  |  |  |  |  |  |  |
| 26 |  |  |  |  |  |  |  |  |  |
| 27 | ESR1 | p.D538G | 456 | 26 | 5.7 | 230 | 24 | 10.4 | 1 |
|  | ESR1 | p.E380Q | 3054 | 60 | 2 | 777 | 12 | 1.5 | 0 |
| 28 | PIK3CA | p.E545K | 2274 | 680 | 29.9 | 802 | 261 | 32.5 | 1 |
|  | TP53 | c.673-1G>T | not covered by panel | | | 727 | 228 | 31.4 | 1 |
| 29 | TP53 | p.R273C | 554 | 295 | 53.3 | 307 | 130 | 42.3 | 1 |
| 30 |  |  |  |  |  |  |  |  |  |
| 31 |  |  |  |  |  |  |  |  |  |
| 32 |  |  |  |  |  |  |  |  |  |
| 33 |  |  |  |  |  |  |  |  |  |
| 34 | AKT1 | p.E17K | 462 | 56 | 12.1 | 86 | 15 | 17.4 | 1 |
| 35 | PIK3CA | p.E545K | 1542 | 55 | 3.6 | 570 | 19 | 3.3 | 0 |
|  | PIK3CA | p.E726K | 2147 | 66 | 3.1 | 659 | 34 | 5.2 | 1 |
| 36 |  |  |  |  |  |  |  |  |  |
| 37 |  |  |  |  |  |  |  |  |  |
| 38 | TP53 | tvc.novel.2 | 439 | 4 | 0.9 | 366 | 4 | 1.1 | 0 |
|  | PIK3CA | p.G1049R | 704 | 22 | 3.1 | 157 | 2 | 1.3 | 0 |
|  | PIK3CA | p.Q546K | 595 | 19 | 3.2 | 390 | 14 | 3.6 | 1 |
| 39 | PIK3CA | p.E545K | 874 | 33 | 3.8 | 375 | 19 | 5.1 | 0 |
|  | PIK3CA | p.E726K | 1135 | 26 | 2.3 | 260 | 0 | 0 | 0 |
|  | TP53 | p.R280K | 429 | 26 | 6.1 | 180 | 10 | 5.6 | 0 |
| 40 |  |  |  |  |  |  |  |  |  |
| 41 | PIK3CA | p.E726K | 2535 | 10 | 0.4 | 349 | 0 | 0 | 0 |
|  | PIK3CA | p.N345K | 1777 | 406 | 22.9 | 177 | 27 | 15.3 | 1 |
| 42 | PIK3CA | p.N345K | 906 | 18 | 2 | 220 | 12 | 5.5 | 0 |
| 43 | PIK3CA | p.H1047R | 1970 | 245 | 12.4 | 214 | 33 | 15.4 | 1 |
|  | SMARCA4 | p.F1142L | not covered by panel | | | 282 | 54 | 19.1 | 1 |
| 44 | PIK3CA | p.E542K | 761 | 199 | 26.2 | 234 | 72 | 30.8 | 1 |
|  | CDH1 | p.Q23* | not covered by panel | | | 38 | 4 | 10.5 | 1 |
| 45 | ERBB2 | p.L755S | 190 | 10 | 5.3 | 94 | 5 | 5.3 | 0 |
|  | PIK3CA | p.H1047L | 253 | 19 | 7.5 | 237 | 29 | 12.2 | 1 |
| 46 | PIK3CA | p.H1047R | 1074 | 24 | 2.2 | 121 | 0 | 0 | 0 |
| 47 | PIK3CA | p.N345K | 1164 | 270 | 23.2 | 201 | 63 | 31.3 | 1 |
|  | TP53 | p.R282W | 461 | 4 | 0.9 | 262 | 0 | 0 | 0 |
| 48 | PIK3CA | p.E545K | 274 | 30 | 11 | 494 | 132 | 26.7 | 1 |
| 49 |  |  |  |  |  |  |  |  |  |
| 50 |  |  |  |  |  |  |  |  |  |
| 51 |  |  |  |  |  |  |  |  |  |
| 52 | ERBB3 | p.D297Y | 620 | 11 | 1.8 | 400 | 11 | 2.8 | 0 |
| 53 | PIK3CA | p.E545K | 1076 | 78 | 7.3 | 325 | 19 | 5.8 | 1 |
| 54 |  |  |  |  |  |  |  |  |  |
| 55 | PIK3CA | p.E545K | 1143 | 10 | 0.9 | 379 | 1 | 0.3 | 0 |
| 56 | PIK3CA | p.H1047R | 579 | 9 | 1.6 | 192 | 6 | 3.1 | 0 |
| 57 |  |  |  |  |  |  |  |  |  |
| 58 |  |  |  |  |  |  |  |  |  |
| 59 |  |  |  |  |  |  |  |  |  |
| 60 | PIK3CA | p.H1047R | 1960 | 58 | 3 | 224 | 12 | 5.4 | 1 |
| 61 |  |  |  |  |  |  |  |  |  |
| 62 | TP53 | p.G199V |  |  | NA | 273 | 14 | 5.1 | 1 |
| 63 | PIK3CA | p.H1047R | 1545 | 11 | 0.7 | 293 | 0 | 0 | 0 |
| 64 |  |  |  |  |  |  |  |  |  |
| 65 |  |  |  |  |  |  |  |  |  |
| 66 | TP53 | p.H193R | 1585 | 50 | 3.2 | 634 | 23 | 3.6 | 0 |
| 67 | PIK3CA | p.E542K | 2952 | 1378 | 46.7 | 768 | 314 | 40.9 | 1 |
|  | TP53 | p.P151H | 1006 | 158 | 15.7 | 289 | 46 | 15.9 | 1 |
|  | PIK3CA | p.H1047R | 2460 | 108 | 4.4 | 462 | 11 | 2.4 | 0 |
| 68 | TP53 | p.G244D | 1206 | 437 | 36.2 | 186 | 67 | 36 | 1 |
| 69 | PIK3CA | p.H1047R | 917 | 13 | 1.4 | 193 | 0 | 0 | 0 |
| 70 | PIK3CA | p.H1047R | 1463 | 73 | 5 | 152 | 0 | 0 | 0 |
|  | CDH1 | c. 1565 +1G>A | not covered by panel | | | 56 | 4 | 7.1 | 1 |


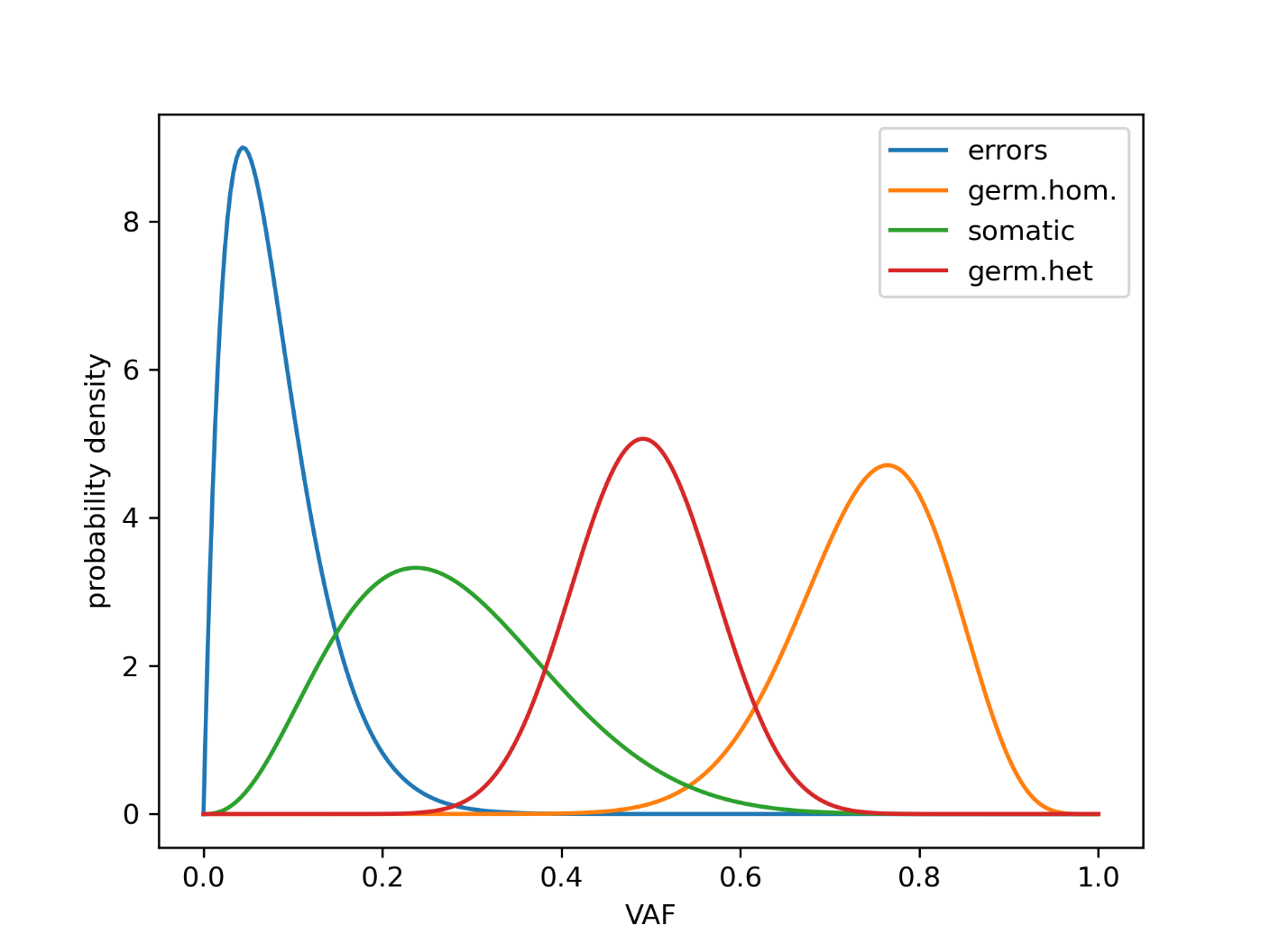


**Figure S1:** Beta distributions of the four expected types of variants. Errors: sequencing errors, germ.hom: germline heterozygous variants, somatic: somatic variants, germ.het: germline homozygous variants.


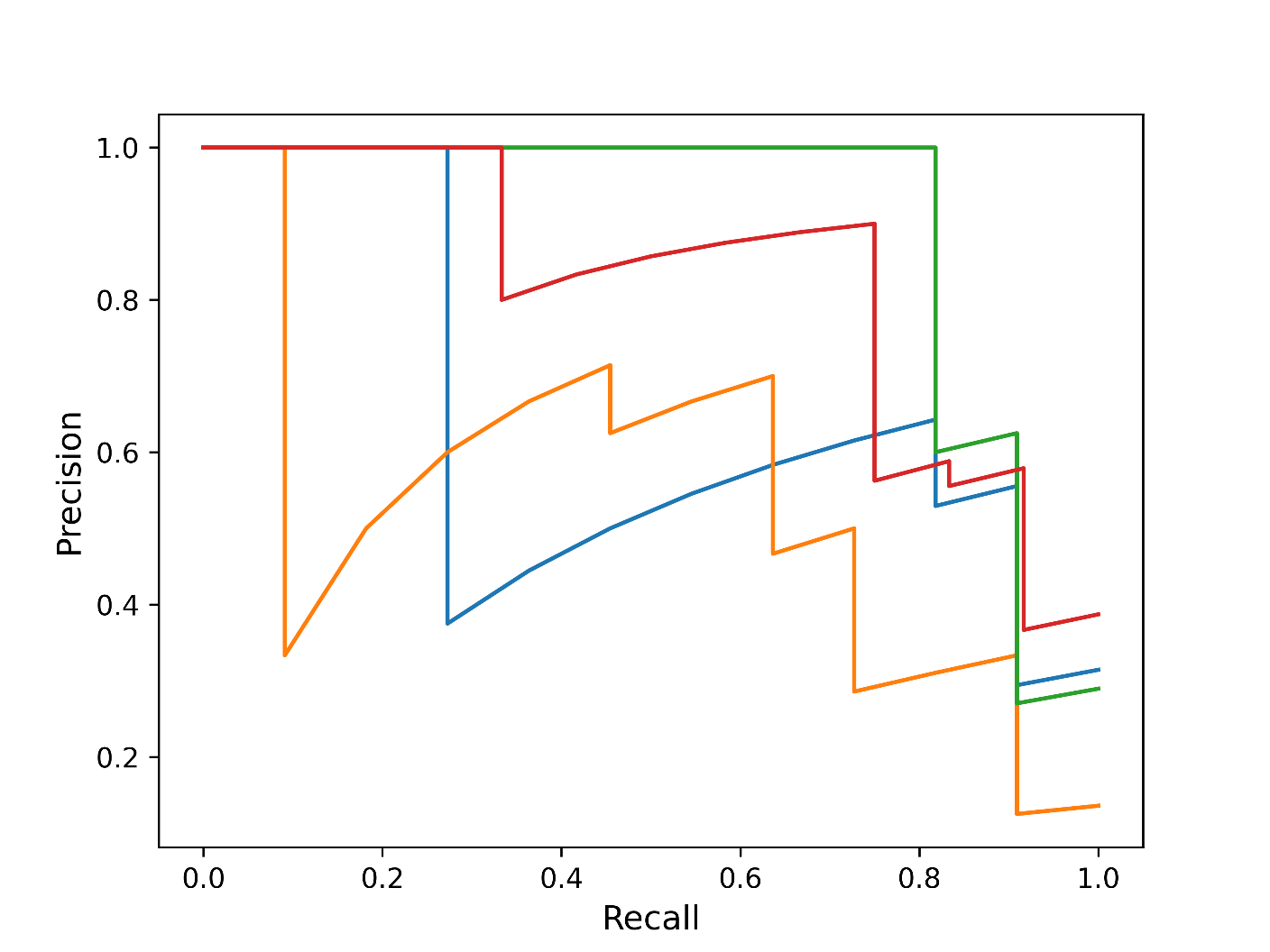


**Figure S2** Precision-recall curves of our SVM model using 4-fold cross validation.

**
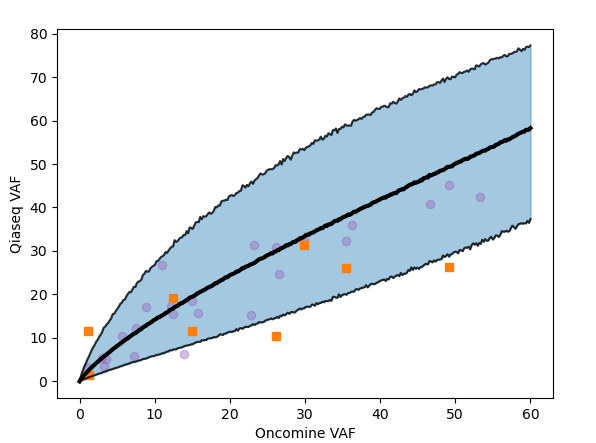
**

**Figure S3** Regression model of mutations called by both panels, predicting the Qiaseq VAF from the Oncomine VAF.

The purple points are the variants which are detected with both the Oncomine and the Qiaseq panel. The orange squares are the patients with an Oncomine mutation and a different Qiaseq mutation: the x coordinate is the Oncomine VAF in that patient and the y coordinate is the Qiaseq VAF of the extra Qiaseq mutation. The fitted model is shown as a solid black line. The blue shaded area shows the 95% posterior interval, which means that for a given Oncomine VAF, if you profiled the same patient with Qiaseq, the Qiaseq VAF would be in the shaded region 95% of the time.


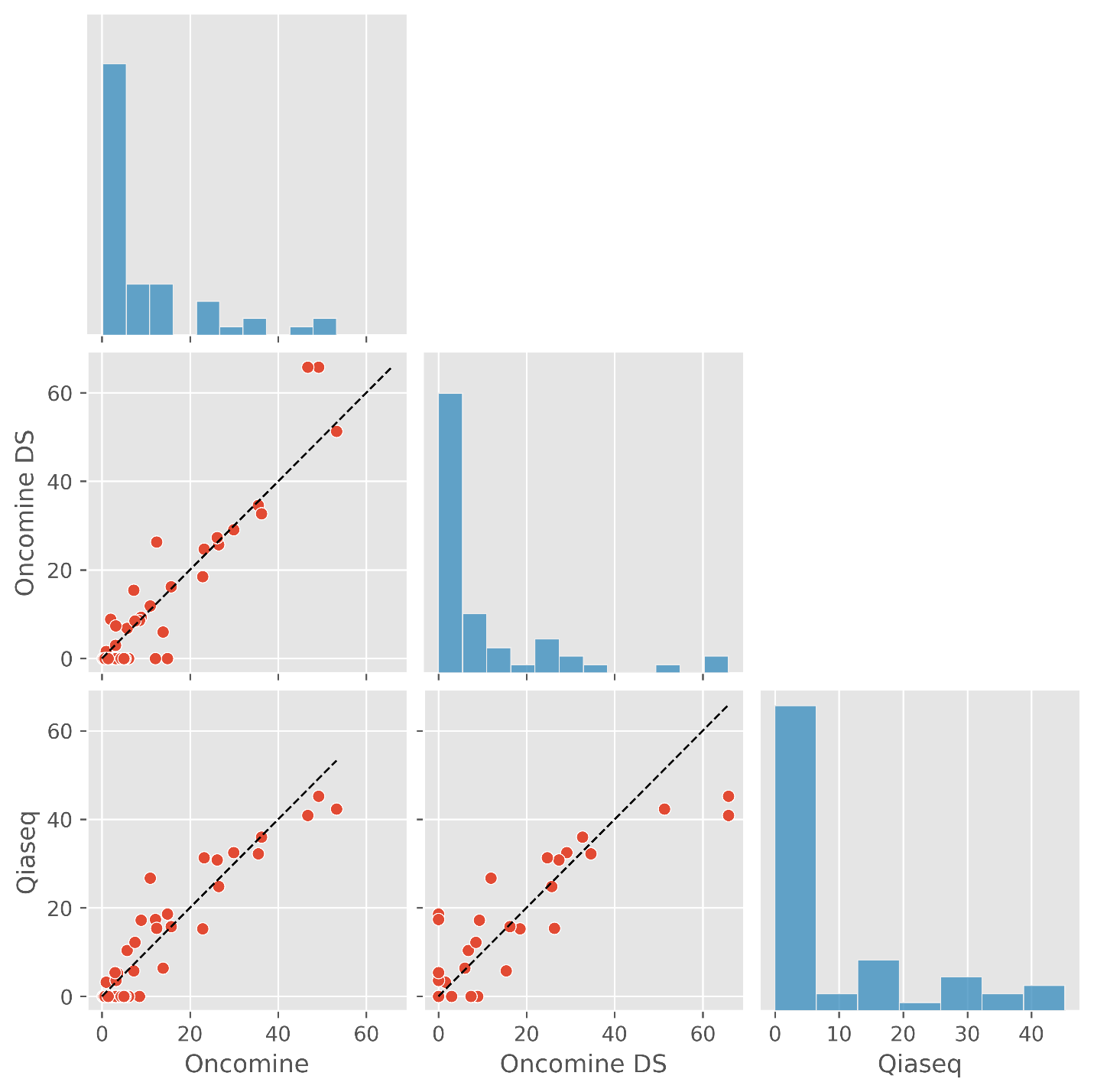


**Figure S4**: Comparison of the VAFs for mutations detected in the common regions between Qiaseq and Oncomine. Plots along the diagonal show the VAF distributions for Oncomine, down-sampled Oncomine (Oncomine DS) and Qiaseq. In off-diagonal plots, each red dot corresponds to a mutation and its *x* and *y* co-ordinates represent the VAF of that mutation in the corresponding panel. The dashed black line show the *y=x* line. For mutations that were not detected we set the VAF to zero.

| 1. 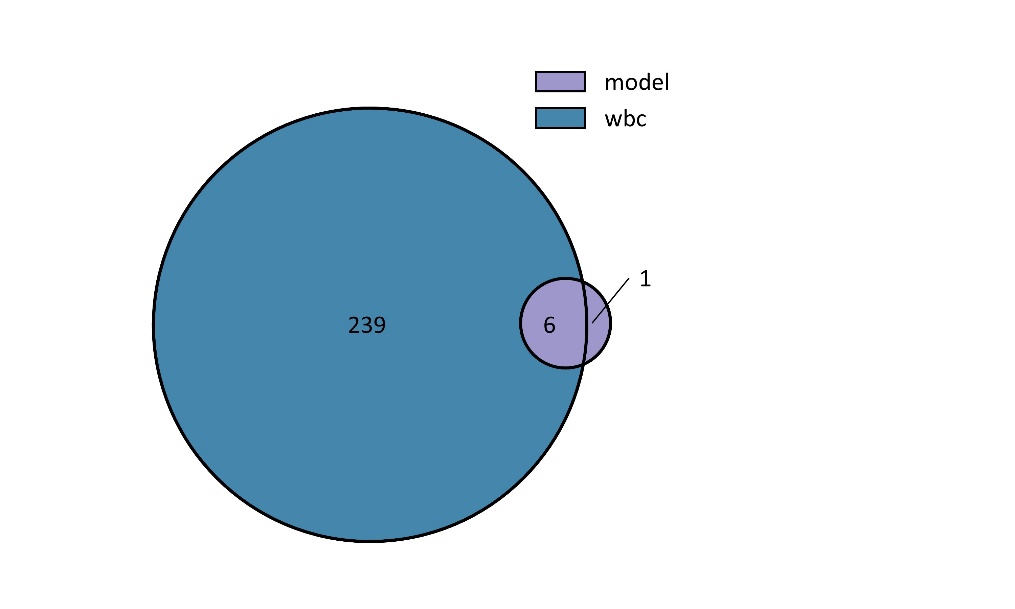 | 1. 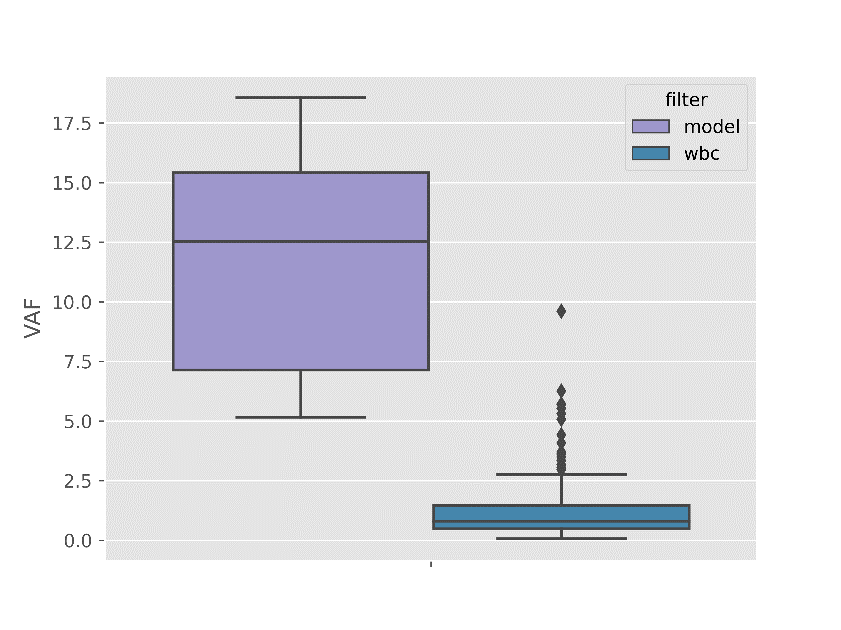 |
| --- | --- |
| **Figure S5:** Comparison of model-based filtering of Qiaseq variant calls to the use of matched germline data from white blood cells. (A) Venn diagram showing the numbers of variants detected using the two approaches. (B) Comparison of the VAF distributions of those variants. | |
